# The effect of IGF-1 on cartilage injury in bone marrow mesenchymal stem cells through the BMP2-Smad1/5 signaling pathway

**DOI:** 10.1007/s11626-025-01015-4

**Published:** 2025-03-05

**Authors:** HuiYue Ye, Liang Shao

**Affiliations:** 1 Department of Traditional Chinese Medicine Orthopedics, Ruian Traditional Chinese Medicine Hospital, No. 498 Anyang Road, Ruian City, 325200 Wenzhou China; 2 Department of Traditional Chinese Medicine Orthopedics, Hangzhou Fuyang Hospital of TCM Orthopedics and Traumatology, Hangzhou, 311400 Zhejiang China; 3 Traditional Chinese Medicine Orthopedics, South Entrance 2, No. 2318 Yuhangtang Road, Hangzhou, 311499 Zhejiang China

**Keywords:** Bone marrow mesenchymal stem cells, Osteoarthritis, Chondrocytes, Insulin-like growth factor-1, Bone morphogenetic protein 2, Smad1/5

## Abstract

The objective of this study is to analyze the effect of insulin-like growth factor-1 (IGF-1) in bone marrow mesenchymal stem cells (BMSCs) on cartilage injury and explore the regulatory mechanism of IGF-1 on the bone morphogenetic protein 2 (BMP2)-Smad1/5 signaling pathway. We cultivated rat BMSCs in vitro and observed their cell morphology using an inverted microscope. Flow cytometry was used to identify the surface antigen expression of BMSCs. IL-1β is used to induce rat chondrocyte ATDC5 to construct a cartilage injury model. We integrated IGF-1 overexpressed BMSCs, empty vector transfected BMSCs, and BMSCs with IL-1, respectively. IL-1β-induced ATDC5 cells were co-cultured for 24 h. We recorded them as BMSCs + IGF-1 group, BMSCs + empty vector group, BMSCs group, and normal cultured ATDC5 cells as the control group. qRT-PCR and Western blot were used to detect IGF-1 mRNA and protein levels in each group. CCK-8 experiment and flow cytometry were used to detect cell proliferation and apoptosis in each group. ELISA is used to detect the levels of TNF-α, IL-8, and IL-6. Western blot was used to detect protein levels of Bax, Bcl-2, Cleaved Caspase-3, Aggrescan, Col II, MMP-1, MMP-13, BMP2, and p-Smad1/5 in each group. Fifty rats were randomly divided into a control group, a model group, a BMSCs group, a BMSCs + empty body group, and a BMSCs + IGF-1 group using a random number table method, with 10 rats in each group. We evaluated cartilage repair using the O’Driscoll scoring system and Mankin’s scoring system. HE staining was used to observe pathological changes in cartilage tissue. qRT-PCR and Western blot were used to detect the expression levels of cartilage repair–related genes OC, GSK-3β, and Runx2 in various cartilage tissues. Overexpression of IGF-1 in BMSCs could enhance IL-1β-induced ATDC5 cell survival rate and the protein level of Bcl-2; reduce apoptosis rate and the protein levels of Bax and Cleaved Caspase-3; decrease the levels of IL-6, TNF-α, and IL-8; increase the protein levels of BMP2, p-Smad1/5, Aggrescan, and Col II; and reduce the protein levels of MMP-1 and MMP-13 (*P* < 0.05). Compared with the model group, the O’Driscoll score in the BMSCs group, the BMSCs + empty body group, and the BMSCs + IGF-1 group was increased; Mankin’s score was decreased; and the expression levels of OC, GSK-3β, and Runx2 were decreased (*P* < 0.05). Compared with the BMSCs group and BMSCs + empty body group, the O’Driscoll score in the BMSCs + IGF-1 group was increased, Mankin’s score was decreased, and the expression levels of OC, GSK-3β, and Runx2 were decreased (*P* < 0.05). Overexpression of IGF-1 in BMSCs could inhibit IL-1β-induced chondrocyte apoptosis, promote cell proliferation, reduce the secretion of inflammatory factors, alleviate chondrocyte damage, and promote cartilage tissue repair. Its mechanism may be related to the activation of the BMP2-Smad1/5 signaling pathway.

## Introduction

Osteoarthritis is a common degenerative bone and joint disease in clinical practice. The incidence rate of osteoarthritis in our country is about 15%. Clinical manifestations include the destruction/degeneration of articular cartilage and osteophyte formation. The pathological characteristics include the loss of extracellular matrix, fibrosis, and degeneration of articular cartilage. Clinically, non-steroidal anti-inflammatory drugs and other treatments are commonly used, but the therapeutic effects are limited. Additionally, the lifespan of prostheses after knee replacement is limited (He *et al*. 2020; Wang *et al*. 2022). Therefore, the repair of articular cartilage damage and the search for treatment schemes for osteoarthritis have become key points in clinical research. Bone marrow mesenchymal stem cells (BMSCs) are mesenchymal cells derived from the bone marrow, which can differentiate into various types of tissues such as cartilage and bone, self-renew, and produce immune regulatory responses. They can also regulate the local microenvironment of cartilage damage, inhibit apoptosis of chondrocytes, and promote tissue repair, but the specific mechanism of BMSCs in treating osteoarthritis is not yet clear (Yu *et al*. 2022). Insulin-like growth factor-1 (IGF-1) is a paracrine growth factor of BMSCs. An increase in its levels can promote the proliferation of chondrocytes, promote cell survival in spinal cord injury, participate in the development and maturation of cartilage, and induce the differentiation of BMSCs into osteoblasts, playing an important role in bone repair and reconstruction (Wu *et al*. 2022; Collins et al. 2023). Previous studies have shown that the activation of the bone morphogenetic protein 2 (BMP2)-Smad1/5 signaling pathway can promote the development, maturation, and differentiation of osteoblasts of cartilage, and participate in the healing and reconstruction of fractures (Nagasaki *et al*. 2023). However, it is not clear whether IGF-1 can regulate the BMP2-Smad1/5 signaling pathway to participate in the process of chondrocyte damage. Therefore, this study mainly explores the impact of overexpression of IGF-1 in bone marrow mesenchymal stem cells on cartilage damage and analyzes its regulatory effect on the BMP2-Smad1/5 signaling pathway.

## Materials and methods

### Morphology and identification of BMSCs

BMSCs (Wuhan Punosei Life Technology Co., LTD, Wuhan, China) were cultured with 90% α-MEM + 10% fetal bovine serum, and then passed through culture when the cells grew to 85% fusion. BMSCs were collected by flow antibody staining, stained with oil red and alizarin red, respectively, and the expression of antibodies CD73, CD90, CD105, CD34, and HLA-DR (BD Company, Franklin Lakes, NJ) on the surface was detected by flow cytometry to identify BMSCs.

### Cell experimental grouping

The third-generation BMSCs were inoculated on 6-well plates (2 × 10^5^ cells/well), and when the cell density reached 60%, empty vectors and IGF-1 overexpression vectors (Shanghai Gima Pharmaceutical Technology Co., LTD, Shanghai, China) were transferred into 6-well plates with Lipofectamine 3000 transfection reagent (Shanghai Gima Pharmaceutical Technology Co., LTD), and then mixed and cultured, and culture medium was changed for 72 h, and IGF-1 expression levels in each group were detected. Chondrocytes ATDC5 (Wuhan Punosei Life Technology Co., LTD) were inoculated in 25 cm^2^ cell culture dishes, and when the cell density grew to 60%, they were inoculated in 24-well plates (1 × 10^4^ cells/well), and 5 ng/mL IL-1β (R&D Systems, Inc., Minneapolis, MN) was used to intervene ATDC5 cells (Li *et al*. 2020), which was recorded as the IL-1β group. BMSCs without transfection, BMSCs transfected with empty vector, and BMSCs transfected with IGF-1 overexpression vector were added and recorded as the BMSCs group, BMSCs + empty vector group, and BMSCs + IGF-1 group, respectively. The normal cultured ATDC5 cells were recorded as the control group, and the cells in each group were cultured in the incubator at 37℃ for 24 h. Cells were collected and subsequent experiments were performed.

### Detecting the cell proliferation by CCK-8 assay

ATDC5 cells of the “2.2.2” group were inoculated into 96-well plates (1 × 10^6^ cells/well), each well was added with 10 μL CCK-8 solution (BD Company), and cultured in a 37℃ incubator for 24 h, and the absorbance value (OD 450 nm) of each hole was detected by an enzyme-labeled analyzer.

### Detecting the apoptosis

The ATDC5 cells in each group “2.2.2” were collected and washed with pre-cooled PBS, centrifuged for 10 min (12,000 r/min, centrifugation radius 8 cm), the supernatant was discarded, the cell precipitation was added with 500 μL binding buffer, and then added with 5 μL Annexin V-FITC and 5 μL PI, respectively (Beijing Solarbio Company, Beijing, China). The apoptosis rate was detected after 10 min at room temperature.

### Detecting the inflammatory factors

The culture medium of ATDC5 cells in each group of “2.2.2” was collected, and the supernatant was extracted by centrifugation at 1500 r/min for 10 min (centrifugation radius 10 cm). The levels of IL-6, TNF-α, and IL-8 in the supernatant were detected by ELISA (Shanghai Enzyme-Linked Biological Company, Shanghai, China).

### Detecting the expression of IGF-1 mRNA

ATDC5 cells in each group of “2.2.2” were taken and 1 mL TRIzol reagent (Thermo Fisher Scientific Company, Waltham, MA) was added to extract total RNA. The reverse transcription system was as follows: RNA 2 μL, 10 × RT buffer 2 μL, dNTP 0.4 μL, Multiscripe 1 μL, 10 × Random Primer 2 μL, RNase-Free ddH2O complement system to 20 μL. The reaction system was prepared according to the qRT-PCR detection kit. IGF-1 mRNA expression was detected by fluorescence quantitative PCR using GAPDH as the internal reference. IGF-1 forward primer 5′-GCTCTTCAGttCGtgTGTG-3′ and reverse primer 5′-GTCTTGGGCATGTCAGTGTG-3′; GAPDH forward primer 5′-ACAGCAACAGGGTGGTGGAC-3′ and reverse primer 5′-TTTGAGGGTGCAGCGAACTT-3′ were designed and synthesized by Shanghai Shenggong Biological Engineering Co., LTD (Shanghai, China).

### The expression of IGF-1, Bax, Bcl-2, Cleaved Caspase-3, Aggrecan, ColII, MMP-1, MMP-13, and BMP2-Smad1/5 signaling pathways were detected by Western blot

ATDC5 cells in each group of “2.2.2” were added with 200 μL lysate to extract total protein; the protein concentration was detected by the BCA method; and the same amount of total protein was added to the sample, separated by SDS-PAGE, transferred to polyvinylidene fluoride membrane, rinsed with 5% skim milk powder, and closed at room temperature for 1 h after TBST. IGF-1 (1:800), Bax (1:500), Bcl-2 (1:500), and Cleaved Caspase-3 (1:500) were added, respectively. Aggrecan (1:800), Col II (1:800), MMP-1 (1:800), MMP-13 (1:800), BMP2 (1:1000), phosphorylated Smad1/5 (p-Smad1/5) (1:1000), Smad 1/5 (1:1000) primary antibody working solution, and internal ginseng GAPDH working solution (1:2000) were incubated overnight at 4℃. After TBST rinse, goat anti-rabbit IgG working solution labeled with horseradish peroxidase was added (1:5000) and incubated at 37℃ for 1 h, and diaminobenzene was colored and exposed in the dark room. Bio-Rad analysis system was used to scan and analyze the relative protein expression. Bax, Bcl-2, Cleaved Caspase-3, Aggrecan, Col II, MMP-1, and MMP-13 were from Abcam Company (Cambridge, UK). BMP2, p-Smad1/5, and Smad1/5 were from Santa Cruz (Santa Cruz, CA).

### Construction of rat model and experiment

Fifty rats, 10 wk of age, weight 280 ~ 320 g were from Beijing Weitongda Biotechnology Co., LTD (animal license number: SCXK, 2023–0014; Beijing, China). Feeding conditions: temperature 20 ~ 25℃, constant temperature 50 ± 5%; the experiment was approved by the management committee of our hospital (No.2024-LW-LC-003). All rats were selected and divided into control group, model group, BMSCs group, BMSCs + empty carrier group, and BMSCs + IGF-1 group according to the random number table method, with 10 rats in each group. The rats in the control group were not treated with any treatment and were cultured normally, and the other rats in the other groups were treated with anterior cruciate ligament resection of the knee joint to establish an osteoarthritis model: After anesthesia, the surgical area was shaved, the medial knee cavity was incised, the patella bone was turned, and the anterior cruciate ligament was cut (Wenjie *et al*. 2022). Criteria for successful modeling (He *et al*. 2019): HE staining showed that the surface of cartilage tissue was rough and thin, some nuclei were shrunk and necrotic, and cell arrangement was disordered. After successful modeling, 10 of them were used as the model group: 0.1 mL normal saline was injected into the joint cavity. In the BMSCs group, 0.1 mL of BMSCs (1 × 10^7^) was injected intra-articularly. The BMSCs + empty vector group was injected with 0.1 mL of empty vector transfected BMSCs (1 × 10^7^). The BMSCs + IGF-1 group was injected with 0.1 mL of BMSCs transfected with IGF-1 overexpression vector (1 × 10^7^).

### Evaluating the degree of cartilage repair and defect

HE staining was used to observe the histopathological changes of cartilage. The knee cartilage tissues of each group were separated, fixed with 10% paraformaldehyde, and then cut into sections after paraffin embedding (thickness was 5 μm). HE staining was used to observe the pathological changes of the cartilage with a microscope. The O’Driscoll score system and Mankin’s score system were used to evaluate cartilage repair. The total score of O’Driscoll score was 24 points, and the higher the score, the closer the cartilage tissue shape was to normal cartilage. Mankin’s score included four aspects: cartilage structure, chondrocytes, matrix staining, and integrity of the tide line. The score of each aspect was 0 to 5 points, and the total score was 20 points. The lower the score, the better the cartilage repair.

### Detecting the cartilage repair–related gene expression level

The expression levels of osteocalcin (OC), glycogen synthase kinase-3β (GSK-3β), and RUN-related transcription factor 2 (Runx2) in the cartilage tissues of rats in the “2.2.9” group were detected by qRT-PCR and Western blot. When detected by qRT-PCR, OC was positive primer 5′-GAGGACCCTCTCTCTGCTCA-3′ and negative primer 5′-GAGCTCACACACCTCCCTGT-3′. GSK—3β—ACCAGAGGCAATCGCACTGTGTAGC forward primer 5′−3′, reverse primer 5′-AATCCGAGCGTGAGGAGGGATAAGG-3′. Runx2 forward primer 5′-TCAGCGtCCTATCAGTTCC-3′, reverse primer 5′-attcaaacGGTTGGAGC-3′, and other experimental steps are the same as “2.2.6.” The working liquid of OC, GSK-3β, and Runx2 (Cell Signaling Technology, Inc., Boston, MA) were all 1:800 when detected by Western blot, and the remaining experimental steps were the same as “2.2.7.”

### Statistical analysis

Statistical software SPSS26.0 was used for analysis, measurement data were expressed as ($\overline{x }$ ± s), independent sample *t-*test was used for comparison between two groups, one-way ANOVA was used for comparison between multiple groups, and LSD-*t* test was used for pairwise comparison. *P* < 0.05 was considered statistically significant.

## Results

### BMSCs form and identification

**Figure 1.**
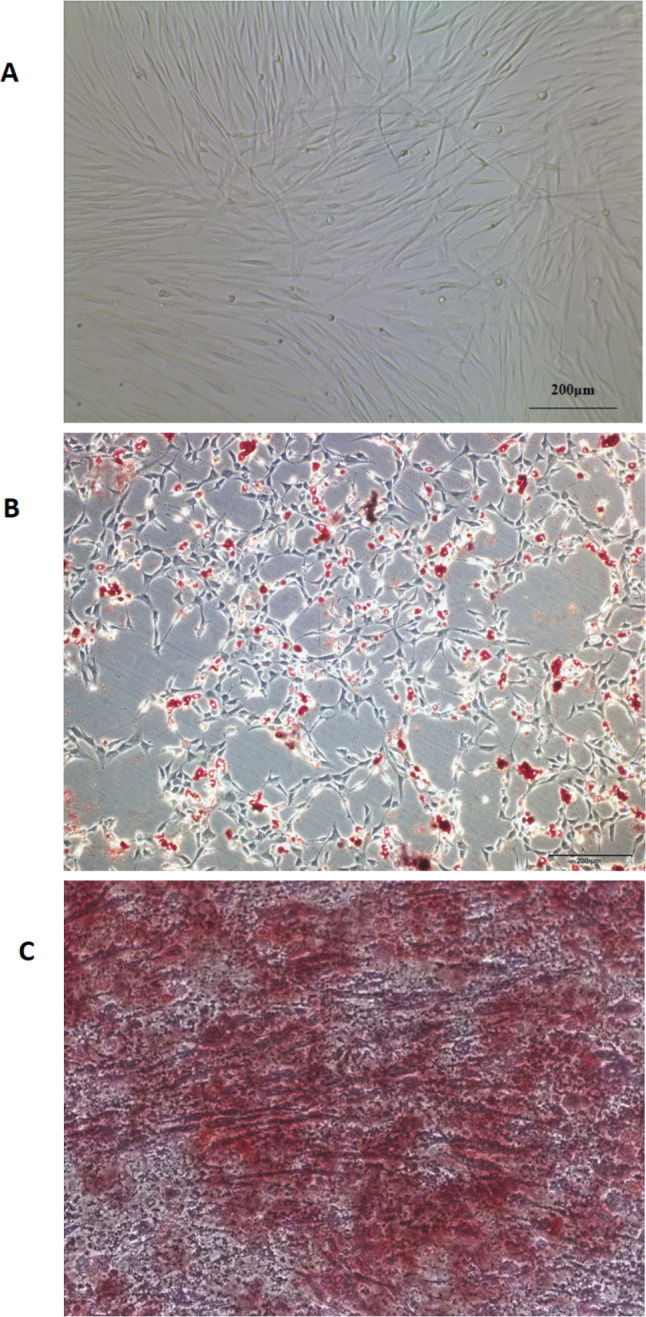
Morphology and induction of BMSCs. (***A***) Adherent cell morphology. (***B***) Lipogenic induction. (***C***) Osteogenic induction.

**Figure 2.**
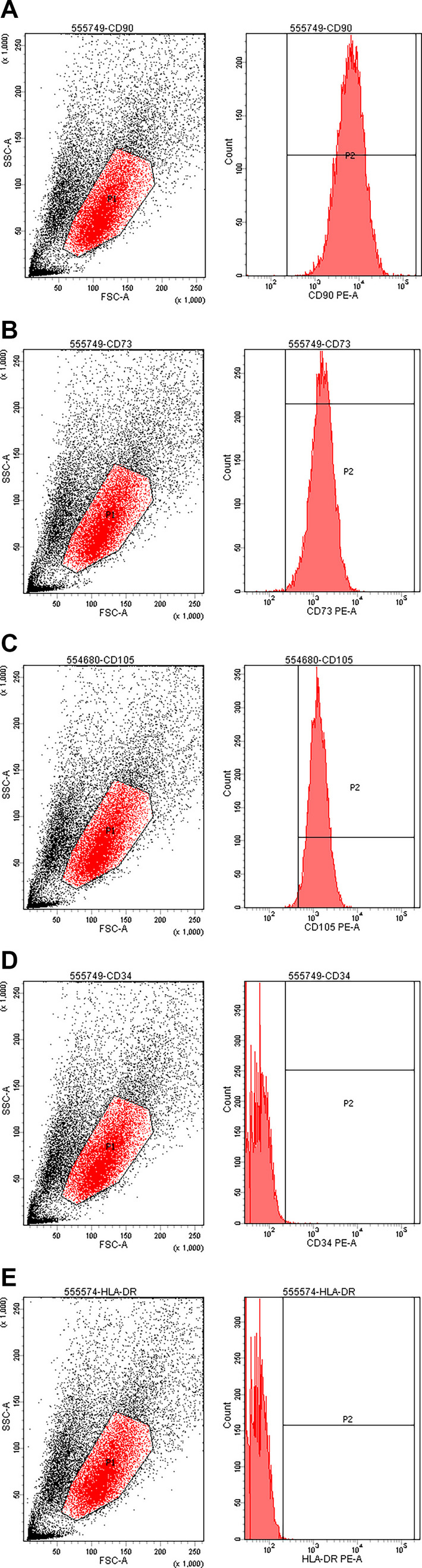
Identification of BMSCs surface antigen expression by flow cytometry. (***A***) CD90( +). (***B***) CD73( +). (***C***) CD105( +). (***D***) CD34(-). (***E***) HLA-DR(-).

After 48 h of fluid exchange, a small number of cells were adherent to the wall and scattered in clumps. The mature BMSCs of the third generation were regularly distributed, with large volume and fusiform nuclei, as shown in Fig. 1. Flow cytometry results showed that the positive rates of CD73, CD90, and CD105 in BMSCs were positive, while CD34 and HLA-DR were negative, which met the phenotype requirements of BMSCs, as shown in Fig. 2.

### Effects of IGF-1 overexpression on proliferation and apoptosis of chondrocytes

**Figure 3.**
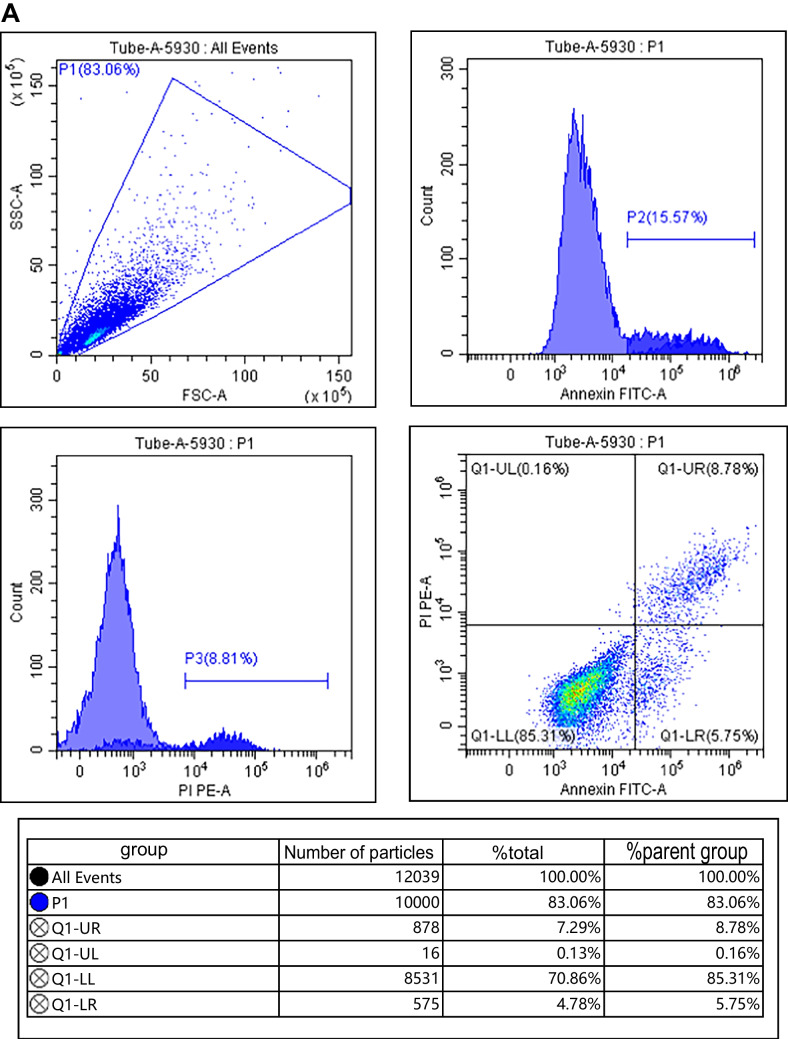

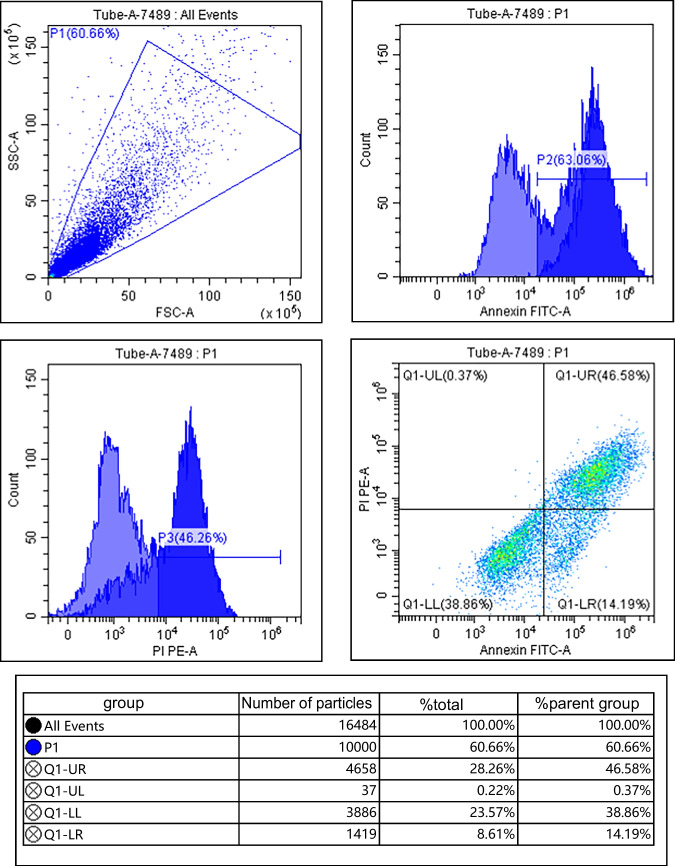

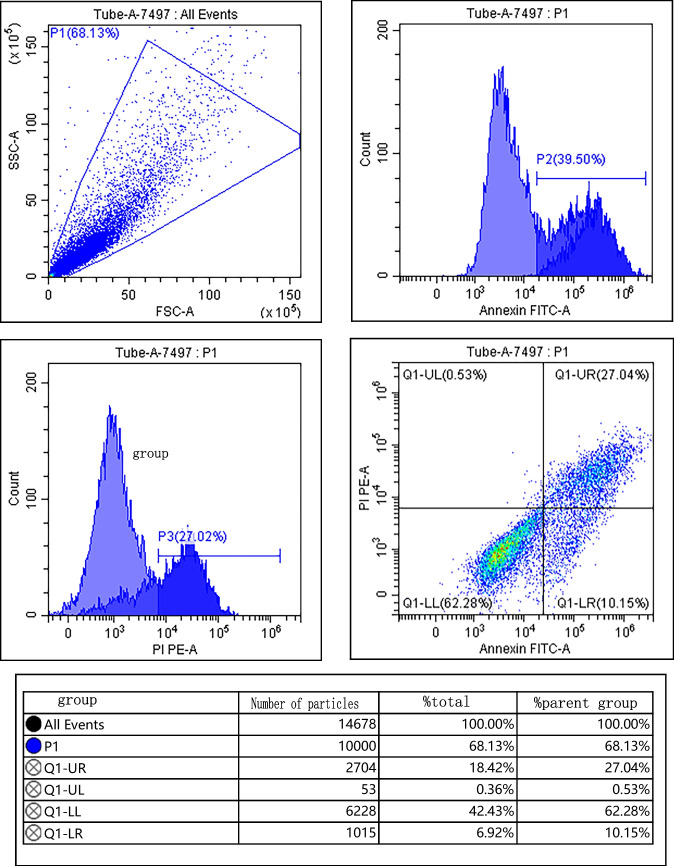

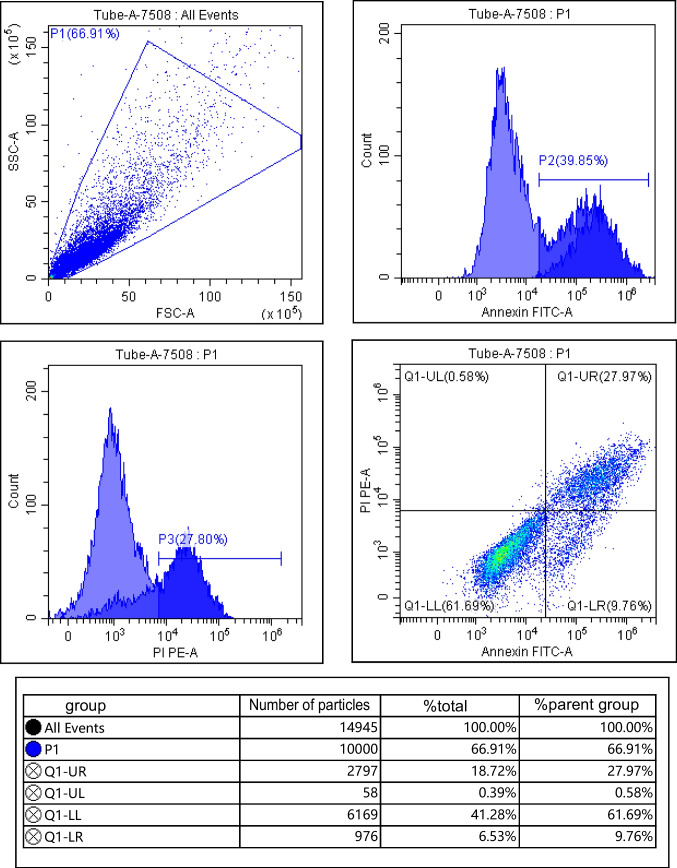

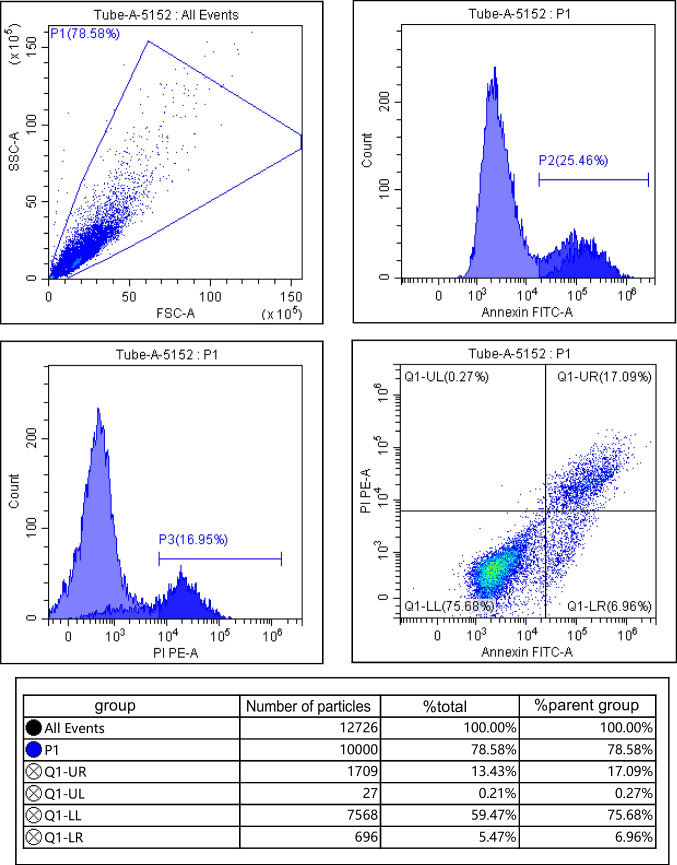

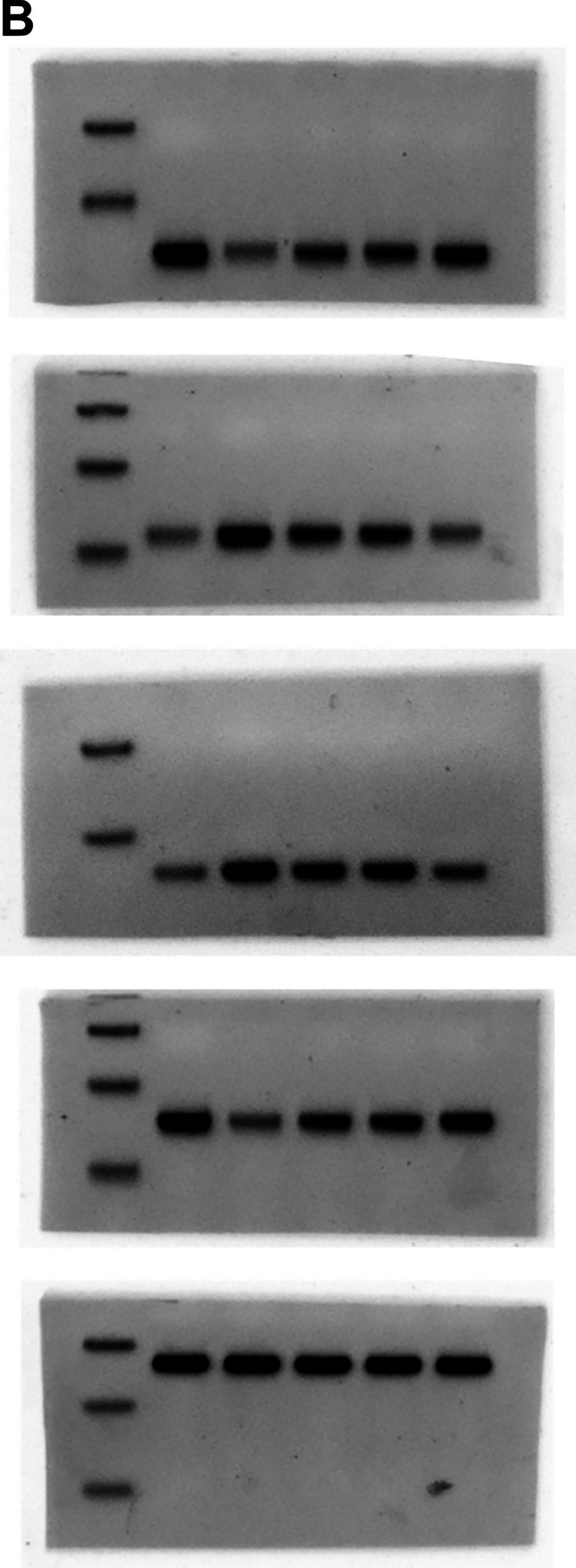
Effect of IGF-1 overexpression on apoptosis of chondrocytes. (***A***) Flow cytometry. (***B***) Apoptotic protein map.

Compared with the control group, IGF-1 mRNA and protein levels in IL-1β group were decreased; cell survival rate was decreased; apoptosis rate and Bax and Cleaved Caspase-3 protein levels were increased; and Bcl-2 protein levels were decreased (*P* < 0.05). Compared with the IL-1β group, IGF-1 mRNA and protein levels in the BMSCs group, BMSCs + empty carrier group, and BMSCs + IGF-1 group were increased; cell survival rate was increased; apoptosis rate and Bax and Cleaved Caspase-3 protein levels were decreased; and Bcl-2 protein levels were increased (*P* < 0.05). Compared with the BMSCs group and BMSCs + empty carrier group, IGF-1 mRNA and protein levels in the BMSCs + IGF-1 group were increased; cell survival rate was increased; apoptosis rate and Bax and Cleaved Caspase-3 protein levels were decreased; and Bcl-2 protein levels were increased (*P* < 0.05), as shown in Fig. 3 and Table 1.

**Table 1.** Effects of IGF-1 overexpression on proliferation and apoptosis of chondrocytes ($\overline{x }$ ± s, *n* = 9)

Groups	IGF-1 mRNA	IGF-1	Cell survival rate (%)	Apoptosis rate (%)	Bax	Cleaved Caspase-3	Bcl-2
Control group	1.00 ± 0.05	0.85 ± 0.16	100.00 ± 0.05	16.32 ± 2.25	0.33 ± 0.05	0.31 ± 0.04	0.93 ± 0.09
IL-1β group	0.28 ± 0.04^*^	0.22 ± 0.08^*^	26.38 ± 6.32^*^	62.25 ± 10.03^*^	0.88 ± 0.11^*^	0.87 ± 0.10^*^	0.24 ± 0.03^*^
BMSCs group	0.63 ± 0.11^*#^	0.52 ± 0.10^*#^	45.58 ± 8.19^*#^	40.21 ± 6.28^*#^	0.53 ± 0.10^*#^	0.50 ± 0.09^*#^	0.49 ± 0.06^*#^
BMSCs + empty carrier group	0.65 ± 0.12^*#^	0.53 ± 0.11^*#^	46.01 ± 9.63^*#^	40.32 ± 6.19^*#^	0.52 ± 0.08^*#^	0.51 ± 0.07^*#^	0.48 ± 0.05^*#^
BMSCs + IGF-1 group	2.01 ± 0.27^*#&@^	0.70 ± 0.13^*#&@^	70.24 ± 10.41^*#&@^	25.96 ± 5.47^*#&@^	0.40 ± 0.07^*#&@^	0.41 ± 0.06^*#&@^	0.68 ± 0.07^*#&@^
*F*	191.404	35.132	117.238	63.724	56.244	71.489	149.018
*P*	0.000	0.000	0.000	0.000	0.000	0.000	0.000

Compared with the control group, ^*^P < 0.05; compared with the IL-1β group, ^#^*P* < 0.05; compared with the BMSCs group, ^&^*P* < 0.05; compared with the BMSCs + empty carrier group, ^@^*P* < 0.05

### Effects of IGF-1 overexpression on levels of inflammatory cytokines in chondrocytes

Compared with the control group, the levels of IL-6, TNF-α, and IL-8 in the IL-1β group were increased (*P* < 0.05). Compared with the IL-1β group, the levels of IL-6, TNF-α, and IL-8 in the BMSCs group, BMSCs + empty carrier group, and BMSCs + IGF-1 group were decreased (*P* < 0.05). Compared with the BMSCs group and BMSCs + empty carrier group, the levels of IL-6, TNF-α, and IL-8 in the BMSCs + IGF-1 group were decreased (*P* < 0.05), as shown in Table 2.

**Table 2.** Effects of IGF-1 overexpression on levels of inflammatory cytokines in chondrocytes ($\overline{x }$ ± s, *n* = 9, pg/mL)

Groups	IL-6	TNF-α	IL-8
Control group	42.26 ± 5.24	83.26 ± 9.24	94.26 ± 11.26
IL-1β group	126.39 ± 9.24^*^	288.64 ± 20.24^*^	388.54 ± 20.24^*^
BMSCs group	88.24 ± 10.03^*#^	200.03 ± 16.21^*#^	216.30 ± 22.06^*#^
BMSCs + empty carrier group	87.54 ± 10.26^*#^	198.54 ± 17.54^*#^	215.27 ± 23.04^*#^
BMSCs + IGF-1 group	60.09 ± 9.57^*#&@^	118.57 ± 20.03^*#&@^	166.41 ± 25.47^*#&@^
*F*	112.240	196.794	240.309
*P*	0.000	0.000	0.000

Compared with the control group, ^*^P < 0.05; compared with the IL-1β group, ^#^*P* < 0.05; compared with the BMSCs group, ^&^*P* < 0.05; compared with the BMSCs + empty carrier group, ^@^*P* < 0.05

### Effects of IGF-1 overexpression on levels of injury-related proteins in chondrocytes

**Figure 4.**
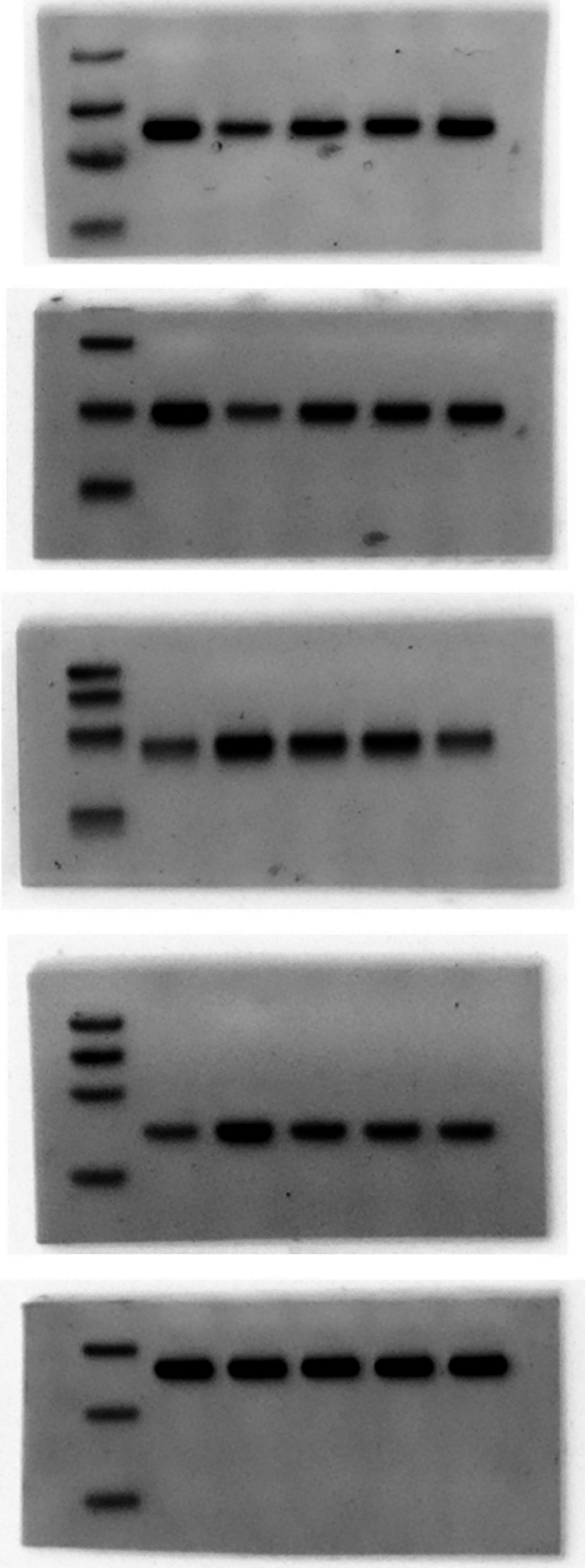
Expression of injury-related proteins in chondrocytes

Compared with the control group, the levels of Aggrecan and ColII protein in the IL-1β group were decreased, while the levels of MMP-1 and MMP-13 protein were increased (*P* < 0.05). Compared with the IL-1β group, Aggrecan and ColII protein levels in the BMSCs group, BMSCs + empty carrier group, and BMSCs + IGF-1 group were increased, while MMP-1 and MMP-13 protein levels were decreased (*P* < 0.05). Compared with the BMSCs group and BMSCs + empty carrier group, Aggrecan and ColII protein levels in the BMSCs + IGF-1 group were increased, while MMP-1 and MMP-13 protein levels were decreased (*P* < 0.05), as shown in Fig. 4 and Table 3.

**Table 3.** Effects of IGF-1 overexpression on levels of injury-related proteins in chondrocytes ($\overline{x }$ ± s, *n* = 9)

Groups	Aggrecan	ColII	MMP-1	MMP-13
Control group	0.96 ± 0.10	0.98 ± 0.11	0.32 ± 0.05	0.22 ± 0.03
IL-1β group	0.31 ± 0.05^*^	0.30 ± 0.06^*^	0.92 ± 0.08^*^	0.90 ± 0.09^*^
BMSCs group	0.55 ± 0.04^*#^	0.57 ± 0.08^*#^	0.60 ± 0.07^*#^	0.62 ± 0.06^*#^
BMSCs + empty carrier group	0.56 ± 0.07^*#^	0.58 ± 0.09^*#^	0.61 ± 0.09^*#^	0.63 ± 0.05^*#^
BMSCs + IGF-1 group	0.67 ± 0.09^*#&@^	0.71 ± 0.10^*#&@^	0.48 ± 0.04^*#&@^	0.44 ± 0.04^*#&@^
*F*	92.242	68.250	93.026	171.162
*P*	0.000	0.000	0.000	0.000

Compared with the control group, ^*^P < 0.05; compared with the IL-1β group, ^#^*P* < 0.05; compared with the BMSCs group, ^&^*P* < 0.05; compared with the BMSCs + empty carrier group, ^@^*P* < 0.05

### Effects of IGF-1 overexpression on levels of BMP2-Smad1/5 signaling pathway–related proteins in chondrocytes

**Figure 5.**
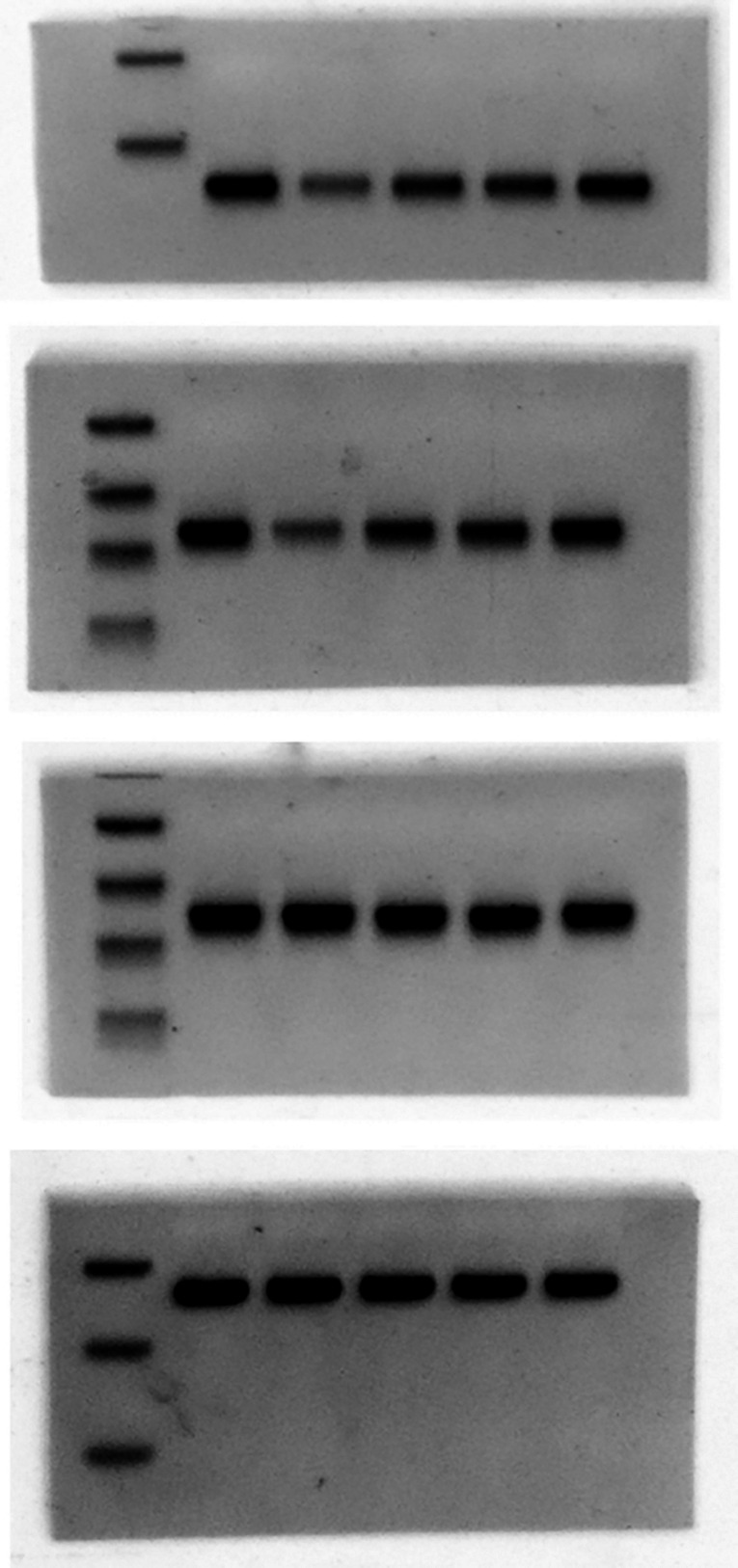
Expression of BMP2-Smad1/5 signaling pathway–related proteins.

Compared with the control group, the levels of BMP2 and P-SMAD1/5 protein in the IL-1β group were decreased (*P* < 0.05). Compared with the IL-1β group, the protein levels of BMP2 and P-SMad1/5 in the BMSCs group, BMSCs + empty carrier group, and BMSCs + IGF-1 group were increased (*P* < 0.05). Compared with the BMSCs group and BMSCs + empty carrier group, the protein levels of BMP2 and P-SMAD1/5 in the BMSCs + IGF-1 group were increased (*P* < 0.05), as shown in Fig. 5 and Table 4.

**Table 4.** Effects of IGF-1 overexpression on levels of BMP2-Smad1/5 signaling pathway–related proteins in chondrocytes ($\overline{x }$ ± s, *n* = 9)

Groups	BMP2	p-Smad1/5	Smad1/5
Control group	0.94 ± 0.10	0.96 ± 0.09	0.88 ± 0.06
IL-1β group	0.24 ± 0.03^*^	0.23 ± 0.01^*^	0.85 ± 0.07
BMSCs group	0.46 ± 0.05^*#^	0.48 ± 0.06^*#^	0.86 ± 0.08
BMSCs + empty carrier group	0.47 ± 0.04^*#^	0.50 ± 0.08^*#^	0.90 ± 0.11
BMSCs + IGF-1 group	0.68 ± 0.06^*#&@^	0.72 ± 0.10^*#&@^	0.89 ± 0.10
*F*	168.919	120.830	0.523
*P*	0.000	0.000	0.719

Compared with the control group, ^*^P < 0.05; compared with the IL-1β group, ^#^*P* < 0.05; compared with the BMSCs group, ^&^*P* < 0.05; compared with the BMSCs + empty carrier group, ^@^*P* < 0.05

### Comparison of cartilage repair and defect degree in each group

**Figure 6.**
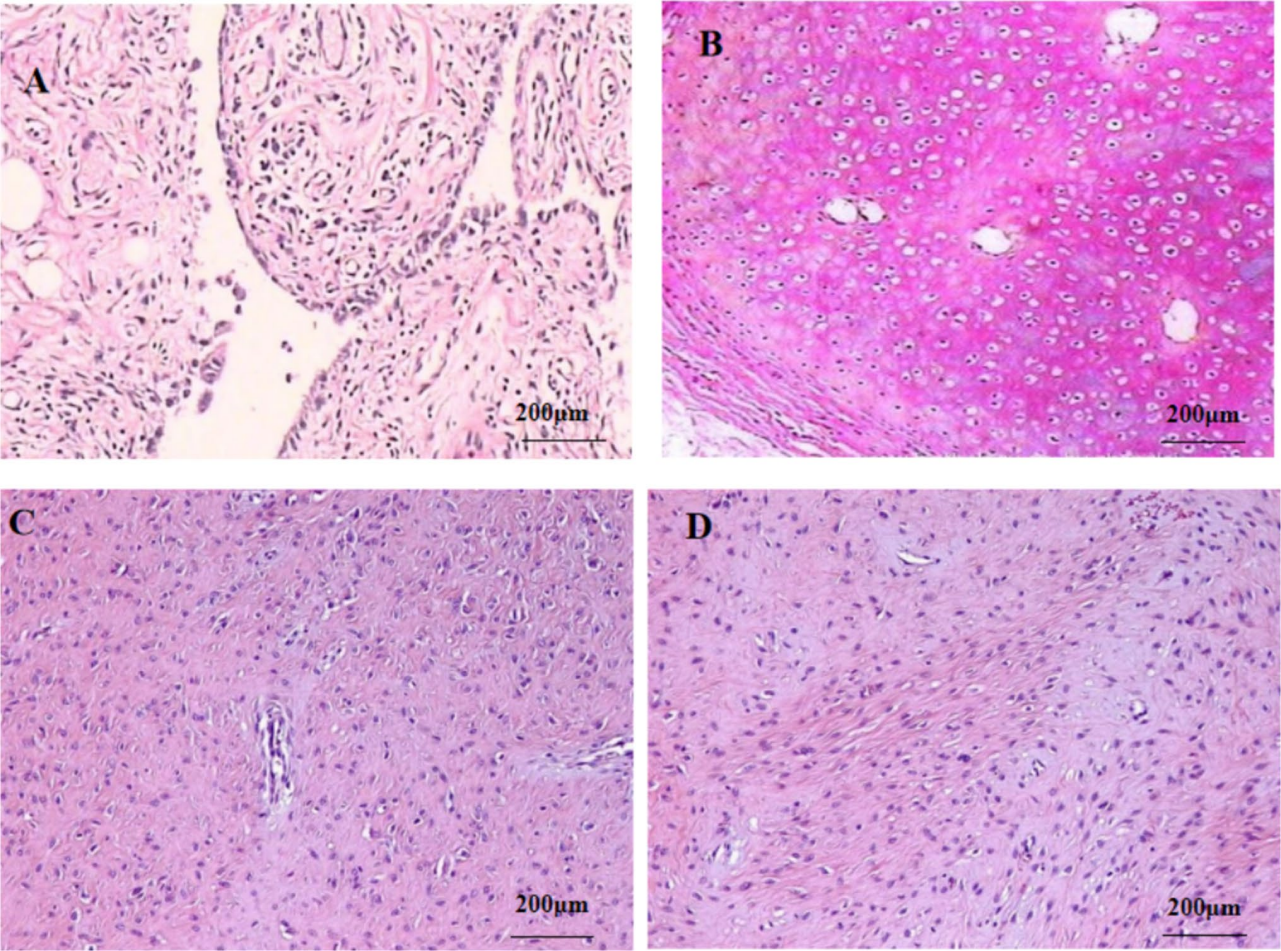
(***A–D***) HE staining results.

In the model group, the articular cartilage surface was uneven, the surface layer was thin, some cells were necrotic, and the cell arrangement was disordered. The degree of articular cartilage degeneration in the BMSCs group and BMSCs + empty carrier group was mild, no signs of defect were found, and the articular surface was relatively flat. In the BMSCs + IGF-1 group, the cartilage was intact without obvious degeneration, and the articular surface was intact, as shown in Fig. 6. Compared with the control group, the O’Driscoll score was decreased and Mankin’s score was increased in the model group (*P* < 0.05). Compared with the model group, the O’Driscoll score in the BMSCs group, BMSCs + empty carrier group, and BMSCs + IGF-1 group was increased, and Mankin’s score was decreased (*P* < 0.05). Compared with the BMSCs group and BMSCs + empty carrier group, the O’Driscoll score was increased and Mankin’s score was decreased in the BMSCs + IGF-1 group (*P* < 0.05), as shown in Fig. 6 and Table 5.

**Table 5.** Comparison of cartilage repair and defect degree of rats in each group ($\overline{x }$ ± s, *n* = 10)

Groups	O’Driscoll score	Mankin’s score
Control group	21.01 ± 1.16	5.26 ± 0.96
IL-1β group	5.02 ± 1.03^*^	16.39 ± 1.46^*^
BMSCs group	8.63 ± 1.15^*#^	10.22 ± 1.03^*#^
BMSCs + empty carrier group	8.70 ± 1.18^*#^	10.28 ± 1.05^*#^
BMSCs + IGF-1 group	16.78 ± 1.09^*#&@^	7.41 ± 1.02^*#&@^
*F*	346.683	140.035
*P*	0.000	0.000

Compared with the control group, ^*^P < 0.05; compared with the IL-1β group, ^#^*P* < 0.05; compared with the BMSCs group, ^&^*P* < 0.05; compared with the BMSCs + empty carrier group, ^@^*P* < 0.05

### Comparison of genes related to cartilage repair in cartilage tissues of rats in each group

**Figure 7.**
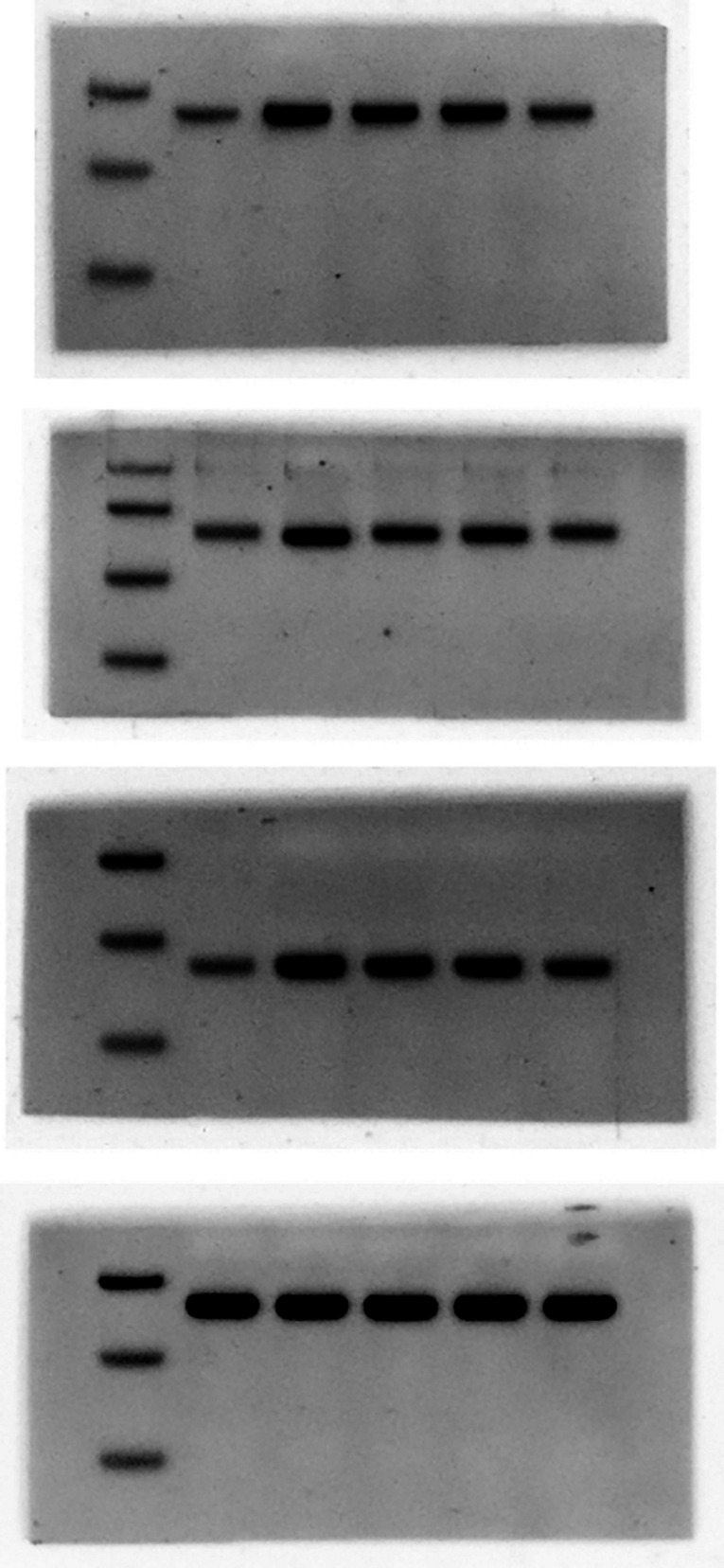
Expression of cartilage repair–related gene protein in cartilage tissue of rats in each group.

Compared with the control group, the expression levels of OC, GSK-3β, and Runx2 in the model group were increased (*P* < 0.05). Compared with the model group, the expression levels of OC, GSK-3β, and Runx2 in the BMSCs group, BMSCs + empty carrier group, and BMSCs + IGF-1 group were decreased (*P* < 0.05). Compared with the BMSCs group and BMSCs + empty carrier group, the expression levels of OC, GSK-3β, and Runx2 in the BMSCs + IGF-1 group were decreased (*P* < 0.05), as shown in Fig. 7 and Table 6.

**Table 6.** Comparison of cartilage repair–related genes in cartilage tissues of rats in each group (± s, *n* = 10)

Groups	mRNA			Proteins		
	OC	GSK-3β	Runx2	OC	GSK-3β	Runx2
Control group	1.00 ± 0.03	1.00 ± 0.05	1.01 ± 0.06	0.32 ± 0.04	0.29 ± 0.03	0.30 ± 0.03
IL-1β group	5.26 ± 0.41^*^	5.41 ± 0.29^*^	4.18 ± 0.31^*^	0.95 ± 0.08^*^	0.94 ± 0.07^*^	0.90 ± 0.10^*^
BMSCs group	3.02 ± 0.33^*#^	3.11 ± 0.35^*#^	2.96 ± 0.24^*#^	0.60 ± 0.07^*#^	0.59 ± 0.06^*#^	0.52 ± 0.09^*#^
BMSCs + empty carrier group	3.01 ± 0.35^*#^	3.12 ± 0.36^*#^	2.94 ± 0.22^*#^	0.58 ± 0.09^*#^	0.57 ± 0.05^*#^	0.53 ± 0.07^*#^
BMSCs + IGF-1 group	2.08 ± 0.05^*#&@^	1.88 ± 0.09^*#&@^	1.74 ± 0.11^*#&@^	0.41 ± 0.03^*#&@^	0.39 ± 0.04^*#&@^	0.44 ± 0.05^*#&@^
*F*	306.376	398.029	344.555	133.265	228.815	93.598
*P*	0.000	0.000	0.000	0.000	0.000	0.000

Compared with the control group, ^*^P < 0.05; compared with the IL-1β group, ^#^*P* < 0.05; compared with the BMSCs group, ^&^*P* < 0.05; compared with the BMSCs + empty carrier group, ^@^*P* < 0.05

## Discussion

Cartilage injury may be related to chondrocyte apoptosis, inflammatory response, reduced cell anabolism, and damage to the integrity of cartilage matrix, etc. Irreversible pathological processes often occur after cartilage injury, resulting in accelerated degeneration of articular surface and subsequent cartilage injury and degeneration. There is still a lack of effective therapeutic methods to reverse cartilage injury in clinic (Ding *et al*. 2021; Li et al. 2022).

BMSCs can be differentiated into various tissues such as cartilage and fat, secrete a variety of cell repair factors, inhibit inflammation, promote bone tissue regeneration, repair knee cartilage injury, and treat osteoarticular cartilage degeneration and other diseases. However, the survival rate and mobility of BMSCs after transplantation are affected, resulting in limited clinical application (Liu and Liu 2021; Wang *et al*. 2021a, b). Therefore, to explore the mechanism of action of BMSCs in alleviating cartilage injury and promoting bone reconstruction is helpful for clinical research and development of new therapeutic schemes. The results of this study showed that the positive rates of CD90 and CD34 in BMSCs were 98.02% and 0.03%, which met the phenotype requirements of BMSCs, and the survival rate of ATDC5 cells induced by IL-1β was decreased, and the apoptosis rate was increased, while BMSCs treatment could improve the cell survival rate and decrease the apoptosis rate. Jiankun *et al*. (2022) showed that BMSCs can regulate the KDM6A/SOX9 signaling pathway to improve cartilage injury in rats with knee arthritis, which can support the conclusion of this study from the side. IGF-1 can promote the proliferation and differentiation of chondrocytes and inhibit cell apoptosis. Previous studies have found that IGF-1 overexpression can maintain the stability of cartilage collagen fibers, promote the proliferation of BMSCs, inhibit inflammation, stimulate the proliferation of chondrocytes, increase the content of extracellular matrix, enhance the activity of osteoblasts, promote the synthesis of type I collagen in bone cells, and reduce collagen degradation (Hossain *et al*. 2021; Wen *et al*. 2021). In this study, it was found that IGF-1 level in ATDC5 cells induced by IL-1β was decreased, while IGF-1 level was increased after BMSCs treatment. Further studies showed that IGF-1 overexpression in BMSCs could significantly improve cell survival rate and reduce apoptosis rate. The Bcl-2 protein family is related to mitochondrial membrane permeability and transferred to mitochondrial outer membrane after stimulated by signal. Bax can enhance mitochondrial membrane permeability and promote cell apoptosis, while Bcl-2 can inhibit cell apoptosis. Cleaved Caspase-3 selectively cleaved key proteins and induced apoptosis (Wang *et al*. 2021a, b). This study found that IL-1β-induced ATDC5 cells increased Bax and Cleaved Caspase-3 protein levels, decreased Bcl-2 protein levels, decreased Bax and Cleaved Caspase-3 protein levels after BMSCs treatment, and increased Bcl-2 protein levels. However, IGF-1 overexpression in BMSCs can further enhance the role of BMSCs, suggesting that IGF-1 overexpression in BMSCs can promote the survival of chondrocytes and inhibit their apoptosis. The reason may be that the overexpression of IGF-1 can activate multiple signal transduction pathways, enhance the ability of BMSCs to resist oxidative stress, and thus improve the effect of BMSCs. Elevated levels of IL-6, TNF-α, and IL-8 can amplify the inflammatory cascade, destroy cartilage tissues/cells, reduce cell proliferation activity, and then participate in the process of cartilage injury (Li *et al*. 2021). The results of this study showed that the levels of IL-6, TNF-α, and IL-8 in BMSCs were significantly reduced after IGF-1 overexpression treatment. Wenyuan *et al*. (2023) showed that BMSCs with IGF-1 overexpression could inhibit the expression of inflammatory factors and alleviate the damage of chondrocytes, which supported the conclusion of this study. It is speculated that the overexpression of IGF-1 can enhance the differentiation ability of BMSCs, inhibit the activity of T lymphocytes and the differentiation of natural killer cells, and thus play an anti-inflammatory role.

Aggrecan and Col II can promote chondrogenesis, and their levels can reflect the injury of chondrocytes. MMP-1 and MMP-13 can degrade extracellular matrix and participate in the process of bone development and bone reconstruction (Zhu *et al*. 2019; Chen *et al*. 2021). Previous studies have shown that the overexpression of IGF-1 can promote the expression of osteogenic stimulating factors and promote the proliferation of osteoblasts, and the reduction of its level can lead to bone anabolic disorders and decrease bone mass (Zhang *et al*. 2019). The results of this study showed that BMSCs can increase the levels of Aggrecan and ColII proteins in IL-1β-induced ATDC5 cells, and reduce the levels of MMP-1 and MMP-13 proteins, while IGF-1 overexpression can enhance the effect of BMSCs on chondrocyte injury–related genes. It is suggested that IGF-1 overexpression can promote the synthesis of extracellular matrix, inhibit the degradation of extracellular matrix, and alleviate the injury of chondrocytes. It is speculated that the overexpression of IGF-1 can accelerate the differentiation of BMSCs, stimulate the secretion of transforming growth factor, inhibit the hypertrophy of mature chondrocytes, and thus promote the repair of cartilage injury. Binding of BMP2 to receptors on the cell membrane can activate intracellular Smad1/5 phosphorylation, promote its action on downstream target genes, activate the transcription of specific target genes, and participate in the process of bone formation (Hurley *et al*. 2022; Zheng *et al*. 2023). Activation of BMP2-Smad1/5 signaling pathway can promote bone formation and osteoblast differentiation, increase the expression level of osteoblast marker genes, and promote bone formation and fracture healing (Jin *et al*. 2023; Hu *et al*. 2024). Le *et al*. (2022) showed that the combined action of IGF-1 and BMP2 could promote fracture healing in fractured rats. However, the regulatory role of IGF-1 and BMP2-SMad1/5 signaling pathway is not yet known. This study found that the protein levels of BMP2 and p-Smad1/5 in BMSCs increased significantly after IGF-1 overexpression, suggesting that IGF-1 overexpression in BMSCs can activate the BMP2-SMad1/5 signaling pathway and reduce cartilage damage. In order to further explore the effect of IGF-1 overexpression in BMSCs on cartilage tissue repair, animal experiments were conducted in this study. The results showed that after IGF-1 overexpression in BMSCs, the O’Driscoll score increased and Mankin’s score decreased, indicating that IGF-1 overexpression can promote cartilage tissue repair and reduce the degree of cartilage tissue damage. Elevated levels of OC, GSK-3β, and Runx2 can regulate the function of osteoclasts, inhibit bone tissue repair, and participate in the occurrence and development of osteoarthritis (Wang *et al*. 2020; Li *et al*. 2023). In this study, it was found that the expression levels of OC, GSK-3β, and Runx2 were decreased after IGF-1 overexpression, suggesting that IGF-1 overexpression may promote cartilage tissue repair by decreasing the levels of OC, GSK-3β, and Runx2. It is speculated that the overexpression of IGF-1 can activate the BMP2-Smad1/5 signaling pathway, promote the expression of transforming growth factor, maintain chondrocyte homeostasis, regulate chondrocyte extracellular matrix synthesis, promote chondrocyte proliferation and microvascular formation, promote the growth and development of bone tissue, and contribute to the repair of cartilage tissue. However, whether IGF-1 has the same stimulating effect on other signaling pathways remains to be confirmed, and the mechanism of action of BMSCs is relatively complex. This study only explored the effect of IGF-1 on cartilage injury/repair, and whether there are other related genes and their specific regulatory mechanisms need to be further explored.

## Conclusion

In summary, overexpression of IGF-1 in BMSCs can promote chondrocyte proliferation, inhibit chondrocyte apoptosis and inflammatory response, alleviate chondrocyte injury, and promote cartilage tissue repair. The mechanism of action may be related to the activation of BMP2-Smad1/5 signaling pathway.

## Data Availability

All data included in this study are available from the corresponding authors upon request.
