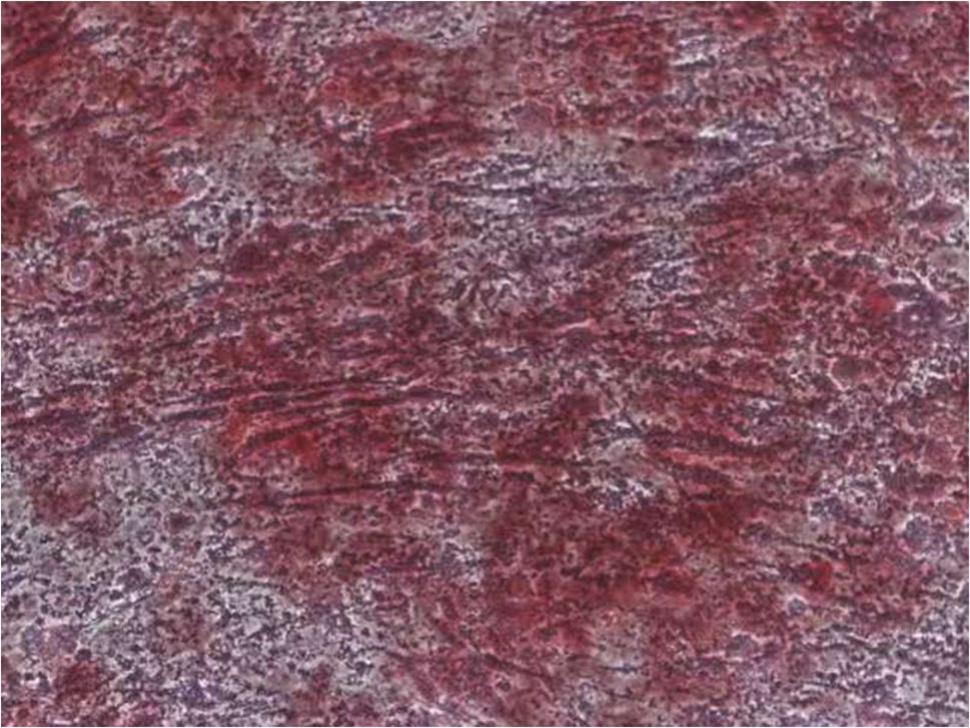# Correction: The effect of IGF-1 on cartilage injury in bone marrow mesenchymal stem cells through the BMP2-Smad1/5 signaling pathway

**DOI:** 10.1007/s11626-026-01179-7

**Published:** 2026-04-07

**Authors:** HuiYue Ye, Liang Shao

**Affiliations:** 1 Department of Traditional Chinese Medicine Orthopedics, Ruian Traditional Chinese Medicine Hospital, No. 498 Anyang Road, Ruian City, 325200 Wenzhou China; 2 Department of Traditional Chinese Medicine Orthopedics, Hangzhou Fuyang Hospital of TCM Orthopedics and Traumatology, Hangzhou, 311400 Zhejiang China; 3 Traditional Chinese Medicine Orthopedics, South Entrance 2, No. 2318 Yuhangtang Road, Hangzhou, 311499 Zhejiang China

**Correction: In Vitro Cellular & Developmental Biology - Animal**

10.1007/s11626-025-01015-4

Following the publication of our manuscript, we have identified the following error.

**Issue Confirmation**

Upon internal review, it was found that an oversight occurred during the archiving of images between collaborative research projects, leading to the inadvertent misuse of an image in Figure 1*C*, resulting in improper duplication with an image used in other related research. This issue stems from a pre-publication image management error.

**Nature of the Issue**

(1) This duplication constitutes unintentional image misuse, with no intent of data fabrication or academic misconduct.

(2) The figure in question serves solely as a morphological example within the text and was not used for any quantitative statistical or data analysis.

(3) All experiments in the paper were strictly conducted using the bone marrow mesenchymal stem cells as declared, with complete and traceable original experimental records and data.

**Impact on Research Conclusions**

(1) This image issue does not affect the scientific conclusions or core findings of this study:

(2) All key conclusions and statistical significance in the paper are based on independent, reproducible quantitative experimental data.

(3) The core mechanism regarding "IGF-1 regulating cartilage repair via the BMP2-Smad1/5 signaling pathway" is jointly confirmed by multiple biological experiments (including qPCR, Western Blot, functional validation, etc.), and its validity does not depend on this example image.

Therefore, the primary scientific contribution of the paper remains unchanged.

The correct image for Figure !c is provided here: